# Prophylactic Management of Radiation-Induced Nausea and Vomiting

**DOI:** 10.1155/2015/893013

**Published:** 2015-09-03

**Authors:** Petra Feyer, Franziska Jahn, Karin Jordan

**Affiliations:** ^1^Department of Radiotherapy, Vivantes Medical Center Berlin-Neukölln, Rudower Straße 48, 12351 Berlin, Germany; ^2^Department of Internal Medicine IV, Hematology and Oncology, Martin-Luther-University Halle-Wittenberg, Ernst-Grube-Straße 40, 06120 Halle (Saale), Germany

## Abstract

The incidence of nausea and vomiting after radiotherapy is often underestimated by physicians, though some 50–80% of patients may experience these symptoms. The occurrence of radiotherapy-induced nausea and vomiting (RINV) will depend on radiotherapy-related factors, such as the site of irradiation, the dosing, fractionation, irradiated volume, and radiotherapy techniques. Patients should receive antiemetic prophylaxis as suggested by the international antiemetic guidelines based upon a risk assessment, taking especially into account the affected anatomic region and the planned radiotherapy regimen. In this field the international guidelines from the Multinational Association of Supportive Care in Cancer (MASCC)/European Society of Medical Oncology (ESMO) and the American Society of Clinical Oncology (ASCO) guidelines as well as the National Comprehensive Cancer Network (NCCN) are widely endorsed. The emetogenicity of radiotherapy regimens and recommendations for the appropriate use of antiemetics including 5-hydroxytryptamine (5-HT_3_) receptor antagonists, steroids, and other antiemetics will be reviewed in regard to the applied radiotherapy or radiochemotherapy regimen.

## 1. Introduction

RINV still can be a debilitating and distressing side effect for patients receiving radiotherapy, which is often underestimated by clinicians. Depending on the site of irradiation, 50–80% of patients undergoing radiotherapy will experience nausea and vomiting. The pathophysiology of RINV is not completely understood, but progress in understanding the pathophysiology and treatment of CINV has greatly influenced that of RINV. Uncontrolled nausea and vomiting can lead to patients delaying or refusing further radiotherapy, thereby compromising their treatment plan [1]. The incidence, classification of risk, and prophylactic management of radiation therapy induced nausea and vomiting (RINV) will be discussed in this paper.

## 2. Pathophysiology

The pathophysiology of radiotherapy-induced nausea and vomiting (RINV) is not well understood but is thought to be similar to that of CINV. The treatment of CINV has therefore guided that for RINV [2]. Progress in understanding the pathophysiology of chemotherapy-induced emesis led to the development of agents that form also the basis for the treatment of RINV.

## 3. Incidence of RINV

As there is a large amount of literature regarding antiemetic therapies to prevent CINV the body of evidence related to RINV is noticeably smaller [3]. Two prospective observational studies provide information on the frequency of RINV and antiemetic measures.

The Italian Group for Antiemetic Research in Radiotherapy (IGARR) analyzed the incidence of RINV in 1020 patients receiving various kinds of RT without concurrent chemotherapy [4]. Overall, nausea and/or vomiting were reported by 28 percent. The median time to the first episode of vomiting was three days. Antiemetic drugs were administered to 17 percent of the patients, including 12 percent treated prophylactically and 5 percent given rescue therapy.

In a second study of 368 patients receiving RT again without concurrent chemotherapy, the overall incidence rates for nausea and vomiting were 39 and 7 percent [5]. Nausea was more frequent in those receiving RT to the lower abdomen or pelvis (66 percent) compared to the head and neck area radiated patients (48 percent).

## 4. Risk Classification

The occurrence of radiotherapy-induced nausea and vomiting (RINV) will depend on radiotherapy-related factors, such as the site of irradiation, dosing, fractionation, irradiated volume, and radiotherapy techniques. Fractionated radiotherapy, for instance, with as many as 40 fractions over a 2-month period, may lead to ongoing, debilitating nausea and vomiting. As a result, patients may delay or refuse and therefore compromise their antineoplastic treatment [4, 5].

### 4.1. Emetogenicity of Radiotherapy

A major difficulty in ensuring effective antiemetic treatment has been the lack of agreement on the emetic potential of different radiotherapy techniques and doses. The extent of irradiation is one of the determinants of risk for RINV. MASCC/ESMO and the ASCO guidelines divide the RINV risk into four categories based upon the radiation field [2, 6, 7] (Table 1).

Intensity-modulated radiation therapy (IMRT), which is becoming the treatment of choice, for example, many head and neck cancers, reduces toxicity by reducing radiation doses to uninvolved normal tissue in the vicinity of tumour targets. However, previously unaffected tissues, such as the brainstem, may receive clinically significant doses that lead to side effects such as nausea and vomiting [8].

Individual patient characteristics also affect the potential for RINV. Apart from those seen in Table 2, general health, concurrent or recent chemotherapy, psychological status, tumour stage, field size, dose per fraction, and overall length of treatment time may all increase or decrease the chance of nausea and vomiting. This is in accordance with the data of the Italian study group. They showed that the statistically significant patient related risk factors were concomitant chemotherapy and a previous experience of vomiting induced by chemotherapy [4].

## 5. Prevention and Treatment

There have been only a few randomized clinical trials evaluating the efficacy of antiemetic drugs for treating RINV. Evidence shows that prevention of these symptoms is better than intervention on an as-needed basis. Of course overtreatment has also to be avoided to prevent side effects coming from the antiemetics themselves. The most studied agents in these settings seem to be 5-hydroxytryptamine (5-HT_3_) receptor antagonists, proving good response and activity. Other agents such as the tachykinin NK-1 receptor antagonist may play a role but these agents need to be further studied in randomized controlled trials.

### 5.1. 5-HT_3_ Receptor Antagonist

The 5-HT_3_ RA class of antiemetics has been used more extensively in clinical practice to treat RINV over the last two decades. Tables 3 and 4 show randomised trials with 5-HT_3_ RAs and/or corticosteroids in patients treated with single or fractionated regimens of radiotherapy. Different compounds and a wide range of doses and schedules were used. Trials in Table 3 reported that, in patients receiving upper abdominal irradiation, 5-HT_3_ RAs provided significantly greater protection against RINV than metoclopramide, phenothiazines, or placebo.

In patients treated with TBI or HBI, 5-HT_3_ RAs provided significantly greater protection against RINV than conventional antiemetics or placebo (Table 4).

Side effects were evaluated by Goodin and Cunningham [9]. The side effects of 5-HT_3_ RAs are usually reported as being mild, with headache, constipation, diarrhoea, and weakness [10–13]. Also 5-HT_3_ RAs sometimes seem to reduce the frequency of diarrhoea, a debilitating side effect of acute enteric radiation toxicity [14, 15].

The 5-HT_3_ RA palonosetron and the transdermal granisetron patch (Sancuso) might be a useful option for patients receiving radiotherapy, although to date only a few studies have been undertaken on their use [1, 16, 17]. Ruhlmann et al. evaluated antiemetic therapy in 48 gynaecological patients receiving fractionated radiotherapy and concomitant weekly cisplatin (40 mg/m^2^) [18]. Antiemetic treatment was palonosetron and prednisolone. Results showed the probability of completing 5 cycles without emesis was 57%. During cycle 1, 42% of patients were nausea-free, but by the fifth cycle only 23% of patients were continuously nausea-free. Half of the patients used rescue remedy at least once during the 5 cycles. The study concluded that palonosetron and prednisolone alone were insufficient antiemetic treatment in these patients and that investigations of the addition of a NK-1 RA to antiemetic treatment under some circumstances would be valuable.

### 5.2. Corticosteroids

Steroids are interesting antiemetic drugs because of their widespread availability, low cost, and reported benefits. One trial has recorded dexamethasone use as a single agent for the prophylaxis of RINV. This double-blind study [19] reported patients who underwent fractionated radiotherapy to the upper abdomen and received either oral dexamethasone (2 mg × 3/day) or placebo during the first week only of a six-week course of radiotherapy. A trial by Wong et al. [20] (Table 3) showed a nonsignificant trend towards improved complete control of nausea (50% versus 38% with placebo) and vomiting (78% versus 71%) (i.e., primary end point was not reached). However, the effects of dexamethasone extended beyond the initial period: complete control of emesis was achieved by significantly more patients over the entire course of radiotherapy (23% versus 12% with placebo) (i.e., secondary end point was reached). Although the study did not show a statistically significant benefit for the primary end point, the results for the secondary end points and quality of life data strongly suggest that the addition of dexamethasone does provide benefits.

As the majority of episodes of nausea and vomiting occur early in the course of radiotherapy, it could be suggested that antiemetics may only be necessary for the first week of treatment [19, 21, 22].

### 5.3. Neurokinin-1 (NK-1) Receptor Inhibitors

The role of NK-1 RAs in the management of CINV is well established; however, neither aprepitant nor fosaprepitant or the newer NK1-RAs have been studied widely in patients with RINV [23]. A useful option in high-risk patients might be the combination of a 5-HT_3_ receptor antagonist and a NK-1 receptor inhibitor. A trial is ongoing which is looking at this combination in radiochemotherapy patients with cervical cancer [24]. Data from a small clinical trial (*n* = 59 patients) presented at the MASCC meeting 2011 provides the first hint that tachykinin NK-1 receptor antagonists in combination with 5-HT_3_ receptor antagonists and dexamethasone proved to be advantageous in the prophylaxis of acute and delayed nausea during simultaneous radiochemotherapy compared with the standard antiemetic treatment. More patients on the emetic prophylaxis containing tachykinin NK-1 receptor antagonists reached a complete response [25]. Dennis et al. showed the combination of granisetron and aprepitant being safe and efficacious for the prophylaxis of RINV in single and multiple fraction moderately emetogenic radiotherapy for thoracolumbar bone metastases evaluating only a very small sample size with 19 patients [26].

### 5.4. Other Agents

Less specific antiemetic drugs, such as prochlorperazine, metoclopramide, and cannabinoids, have been shown to have limited efficacy in the prevention and treatment of RINV, and this is generally in patients with milder symptoms. The use of THC was slightly more beneficial than the use of prochlorperazine [27] but generally shows an inferior safety profile, including sedation and euphoria/dysphoria. Salvo et al. showed in a meta-analysis from 2012 the superiority of 5-HT_3_ receptor antagonist not only to placebo but also to metoclopramide and other antiemetic drugs [28].

### 5.5. Duration of Prophylaxis

The appropriate duration of antiemetic prophylaxis for patients receiving fractionated radiotherapy is not clear. There have been no randomized trials using 5-HT_3_ receptor antagonists that compared a five-day course of treatment with a more protracted course. A systematic review including 25 randomized and nonrandomized trials revealed that 5-HT_3_ receptor antagonists were most commonly administered for the entire duration of a course of radiotherapy. Thus we still lack evidence related to duration and timing of 5-HT_3_ receptor antagonist therapy [1].

Despite this lack of evidence the current guidelines recommended that the decision on whether or not to continue antiemetic prophylaxis beyond the first week should be based upon an assessment of the risk of emesis as well as relevant individual factors. In a recent article it was noted that patients had significantly greater proportions of RINV during the first and second week of treatment [29].

### 5.6. Rescue Therapy

The benefit of 5-HT_3_ receptor antagonists once nausea or vomiting occurs has been suggested in all studies, but there are no trials specifically in this setting [30–32]. The emerging role of olanzapine in breakthrough emesis in patients with CINV has not been studied in RINV yet [33].

However, as the pathophysiology of RINV is thought to be similar to that of CINV, a treatment attempt with olanzapine might be useful.

## 6. Guidelines and Patient Management

The development of new antiemetic agents and a better understanding of their activity as a function of the emetogenicity of different RT regimens have led to the generation of guidelines for patient management. The most recent guidelines were promulgated by the Multinational Association for Supportive Care in Cancer (MASCC) and European Society for Medical Oncology (ESMO) in 2009 [1] which were subsequently endorsed by the American Society of Clinical Oncology (ASCO) in 2011 [7]. A brief summary of these guidelines is presented in Table 5.

For patients at* high risk* of developing RINV (Table 5), a prophylaxis with a 5-HT_3_ receptor antagonist is recommended by the guidelines. Based upon results from patients receiving highly emetogenic chemotherapy, the addition of dexamethasone to the 5-HT_3_ receptor antagonist is suggested.

For patients at* moderate* risk of developing RINV (Table 5), prophylaxis with a 5-HT_3_ receptor antagonist is the prophylaxis of choice. However, in this risk group, the addition of short course dexamethasone to the 5-HT_3_ receptor antagonist may be offered during fractions 1–5.

For patients at* low risk* of developing RINV (Table 5), either prophylaxis with a 5-HT_3_ receptor antagonist or observation with rescue treatment is suggested.

For patients at* minimal risk* of developing RINV (Table 5), a prophylactic antiemetic treatment is not necessary. If patients develop RINV, treatment with a dopamine receptor antagonist or a 5-HT_3_ receptor antagonist might be appropriate.

For patients being treated with* concomitant RT* and chemotherapy, antiemetic prophylaxis should be based upon the higher chemotherapy or RT risk category.

The NCCN guidelines do not follow the separation in four risk categories and are therefore less specific [34].

## 7. Conclusion

Radiation-induced nausea and vomiting (RINV) is a frequent complication of radiation therapy. Its effect on patients' quality of life should not be underestimated, especially as such effects may compromise or delay treatments. Therefore, patients at risk of RINV should always be offered the most effective antiemetic prophylaxis as suggested by the international guidelines. However, it has to be acknowledged that the implementation of these guidelines in practice varies regarding risk estimation and different countries [35, 36]. There is an additional need to investigate the importance of the individual risk factors of patients regarding the duration of antiemetic treatment. Furthermore recent developments have to be taken into account: the role of the new antiemetic agents such as NEPA, a fixed dose combination of netupitant and palonosetron, rolapitant, a new NK-1 RA, and the potential value of rescue medications such as olanzapine and ginger in the RINV setting [37].

## Figures and Tables

**Table 1:** Risk categories adapted from [1].

Emetogenic potential	Risk of emesis without antiemetic prophylaxis	Location
High	Risk > 90 percent	Total-body irradiation (TBI), total nodal irradiation (TNI)
Moderate	Risk 60 to 90 percent	Upper abdominal irradiation, hemibody irradiation (HBI), and upper body irradiation (UBI)
Low	Risk 30 to 60 percent	Cranium (all), craniospinal, head and neck, lower thorax region, pelvis
Minimal	Risk < 30 percent	Breast and extremities

**Table 2:** Individual risk factors adapted from [1].

Risk factor	Risk score
Age	
>55 years	0
<55 years	1
Sex	
Male	1
Female	2
Alcohol consumption	
Yes (>100 g/day)	0
No	1
Previous nausea & vomiting	
Yes	1
No	0
Anxiety	
Yes	1
No	0

Risk profile (4 = normal risk ∣ 5-6 high risk)	

**Table 3:** Randomised clinical trials with 5-HT_3_ RAs and/or steroids in patients undergoing upper abdominal irradiation adapted from [1].

Study	*n*	Radiotherapy regimen	Antiemetic treatment	CR (% of patients)	Result
Priestman et al. (1990) [38]	82	8–10 Gy single fraction	OND 8 mg × 3/day p.o. for 5 days	97	OND better than placebo
			MCP 10 mg × 3/day p.o. for 5 days	46	

Priestman et al. (1993) [12]	135	1.8 Gy/day for at least 5 fractions	OND 8 mg × 3/day p.o.	61	OND better than placebo (for vomiting)
			PCP 10 mg × 3/day p.o.	35	

Franzén et al. (1996) [15]	111	1.7 Gy/day for ≥10 fractions	OND 8 mg × 2/day p.o.	67	OND better than placebo
			Placebo	45	

Bey et al. (1996)^a^ [39]	50	≥6 Gy single fraction	DOL 0.3 mg/kg i.v.	100^b^	DOL better than placebo
			DOL 0.6 mg/kg i.v.	93^b^	
			DOL 1.2 mg/kg i.v.	83^b^	
			Placebo	54^b^	

Aass et al. (1997) [40]	23	2 Gy/day to 30 Gy in 15 fractions	TRO 5 mg/day p.o.	91	TRO better than MCP
			MCP 10 mg × 3/day p.o.	50	

Lanciano et al. (2001) [41]	260	10–30 fractions	GRAN 2 mg/day	57.5	GRAN better than placebo
		(1.8–3 Gy/fraction)	Placebo	42	

Wong et al. (2006) [20]	211	≥15 fractions to the upper abdomen to a dose of 20 or more Gy	OND 8 mg b.i.d. for 5 days + placebo for 5 days	71^c^ 12^d^	OND + DEX better than OND alone
			OND 8 mg b.i.d. + DEX 4 mg for 5 days	78^c^ 23^d^	

Mystakidou et al. (2006) [32]	288	Fractionated radiotherapy of moderate or high emetogenic potential	TRO 5 mg daily starting from 1 day before RT until 7 days after RT	Incidence of vomiting was 2.19 times higher in TRO rescue arm (*P* = 0.001)	Prophylactic TRO better than rescue TRO
			TRO 5 mg on an as-needed basis (rescue)		

Ruhlmann et al. (2013) [18]	48	5 fractions/wk, 1.8–2.0 Gy/fraction on days 0–4 + cisplatin 40 mg/m^2^ on day 1	PAL 0.25 mg + PRED 100 mg o.d. on day 1, plus PRED 50 mg on day 2, and PRED 25 mg on days 3 and 4	During cycle 1, 42% nausea-free, after 5 cycles only 23% nausea-free	PAL & PRED insufficient for this treatment regimen

^a^Dolasetron i.v.: no longer available in the USA (FDA 2010) and not recommended elsewhere (MASCC guidelines 2013).

^b^CR: complete plus major response.

^c^Primary end point: CR days 1–5.

^d^Secondary end point: CR days 1–15.

p.o. = orally; i.v. = intravenously; b.i.d. = twice daily; wk = week; DEX = dexamethasone; DOL = dolasetron; GRAN = granisetron; MCP = metoclopramide; PAL = palonosetron; PRED = prednisolone; OND = ondansetron; PCP = prochlorperazine; TRO = tropisetron.

**Table 4:** Randomised clinical trials with 5-HT_3_ RAs in patients undergoing TBI and HBI adapted from [1].

Study	*n*	Radiotherapy regimens	Antiemetic treatment	CR (% of patients)	Result
Grant Prentice et al. (1995) [11]	30	7.5 Gy TBI single fraction	GRAN 3 mg i.v. versus MCP 20 mg i.v. + DEX 6 mg/m^2^ i.v. + LOR 2 mg i.v.	53 13	GRAN better than MCP + DEX + LOR

Tiley et al. (1992) [42]	20	10.5 Gy TBI single fraction	OND 8 mg i.v. Placebo	90^a^ 50^a^	OND better than placebo

Spitzer et al. (1994) [43]	20	1.2 Gy × 3/day TBI 11 fractions to a total dose of 13.2 Gy	OND 8 mg × 3/day p.o. Placebo	50 0	OND better than placebo

Sykes et al. (1997) [44]	66	8–12.5 Gy HBI single fraction	OND 8 mg × 2 p.o. versus CLP 25 mg × 3 p.o. + DEX 6 mg × 3 p.o.	34	OND better than CLP + DEX

Huang et al. (1995) [45]	116	7–7.7 Gy	OND 8 mg i.v. + DEX 10 mg versus MCP 10 mg + DEX 10 mg	84 20	OND + DEX better than paspertin + DEX

Spitzer et al. (2000) [13]	34	1.2 Gy × 3/day TBI 11 fractions to a total dose of 13.2 Gy	OND 8 mg × 3/day p.o. versus GRAN 2 mg × 1/day p.o.	47 61	No difference

^a^All patients received i.v. dexamethasone (8 mg) and phenobarbitone (60 mg/m^2^).

CLP = chlorpromazine; CR = complete response; DEX = dexamethasone; GRAN = granisetron; HBI = half-body irradiation; LOR = lorazepam; MCP = metoclopramide; OND = ondansetron; TBI = total-body irradiation; p.o. = orally; i.v. = intravenously.

**Table 5:** Key recommendations of antiemetic guideline groups adapted from [1, 7].

Risk category	Dose	Schedule
High emetic risk		
5-HT_3_ receptor antagonist		5-HT_3_ receptor antagonist before each fraction throughout XRT, continued for at least 24 hours after completion of XRT
Granisetron^∗^	2 mg orally; 1 mg or 0.01 mg/kg i.v.	
Ondansetron^∗^	8 mg orally twice daily; 8 mg or 0.15 mg/kg i.v.	
Palonosetron^†^	0.50 mg orally; 0.25 mg i.v.	
Dolasetron	100 mg orally only	
Tropisetron	5 mg orally or i.v.	
Corticosteroid		
Dexamethasone	4 mg orally or i.v.	During fractions 1–5

Moderate emetic risk		
5-HT_3_ receptor antagonist	Any of the above listed agents are acceptable; note preferred options^†^	5-HT_3_ receptor antagonist before each fraction throughout XRT
Corticosteroid		
Dexamethasone	4 mg i.v. or orally	During fractions 1–5

Low emetic risk		
5-HT_3_ receptor antagonist	Any of the above listed agents are acceptable; note preferred options	5-HT_3_ receptor antagonist as either rescue or prophylaxis; if rescue is used, then prophylactic therapy should be given until the end of XRT

Minimal emetic risk		
5-HT_3_ receptor antagonist	Any of the above listed agents are acceptable; note preferred options	Patients should be offered either class as rescue therapy; if rescue is used, then prophylactic therapy should be given until the end of XRT
Dopamine receptor antagonist		
Metoclopramide	20 mg orally	
Prochlorperazine	10 orally or i.v.	

^∗^Preferred agents; ^†^no data are currently available on the appropriate dosing frequency with palonosetron in this setting. The Update Committee suggests that dosing every second or third day may be appropriate for this agent.

5-HT_3_  =  5-hydroxytryptamine-3; i.v., = intravenously; XRT = radiation therapy.
